# Modelling of Diffusion and Reaction of Carbon Dioxide and Nutrients in Biofilm for Optimal Design and Operation of Emerging Membrane Carbonated Microalgal Biofilm Photobioreactors

**DOI:** 10.3390/membranes15090269

**Published:** 2025-09-04

**Authors:** Meilan Liu, Baoqiang Liao

**Affiliations:** 1 Department of Mechanical and Mechatronics Engineering, Lakehead University, 955 Oliver Road, Thunder Bay, ON P7B 5E1, Canada; mliu@lakeheadu.ca; 2 Department of Chemical Engineering, Lakehead University, 955 Oliver Road, Thunder Bay, ON P7B 5E1, Canada

**Keywords:** membrane photobioreactor, membrane carbonated microalgal biofilm photobioreactor, microalgal biofilm, gas-permeable membrane, mathematical modelling, microalgae, nutrient removal, wastewater

## Abstract

The biological performance and carbon dioxide (CO_2_) flux of the novel and emerging concept of a membrane carbonated microalgal biofilm photobioreactor (MC-MBPBR) for wastewater treatment were investigated using mathematical modelling in conjunction with the finite-difference method. A set of differential equations was established to model the performance of an MC-MBPBR. The impacts of CO_2_ partial pressure, wastewater characteristics, and biofilm thickness on the concentration profiles and fluxes of CO_2_ and nutrients (N and P) to the biofilm of the MC-MBPBR were systematically studied. The modelling results showed profound impacts of these parameters on process efficiency (CO_2_ transfer and N and P removals) and the existence of an optimal biofilm thickness for maximum CO_2_, N, and P fluxes into the biofilm. Penetration of CO_2_ through the biofilm into the bulk water phase might occur under certain conditions. An increase in gaseous CO_2_ and increased influent N and P concentrations led to higher CO_2_, N, and P fluxes. The optimal biofilm thickness varied with the change in wastewater characteristics and gaseous CO_2_ concentration. The modelling results were in relatively good agreement with experimental results from the literature. The proposed mathematical models can be used as a powerful tool to optimize the design and operation of the novel MC-MBPBR for wastewater treatment and microalgae cultivation.

## 1. Introduction

In recent years, microalgae have attracted increasing interests, due to their high productivity of many valuable bioproducts and biofuels, their capability for nutrient (N and P) uptakes from wastewater, and their ability to abate greenhouse gas (CO_2_) from the atmosphere and industrial waste gases [1,2]. The global algal economy had annual revenues of USD 7 to 8 billion [3]. However, the cell concentration of suspended microalgae culture is dilute and usually less than 0.5–2 g/L [3]. Conventionally, microalgae have been cultivated in open ponds or closed photobioreactors, both requiring large volumes of water and a significant amount of energy input for microalgal cultivation, harvesting, and dewatering [2,3,4,5,6]. Current technologies employed to concentrate microalgae biomass include coagulation, flocculation, flotation, centrifugation, filtration, and gravity sedimentation [3,7]. These processes are, however, associated with the use of chemicals and/or high energy consumption [3,5,7]. It has been estimated that 90% of the equipment cost relates to the dewatering of microalgal biomass [8,9], while the harvesting of microalgal biomass from the culture medium represents 20–30% of the total cost of microalgae production [10]. This high operational cost is deemed a bottleneck for the commercialization of large-scale microalgae production [7].

**Figure 1:**
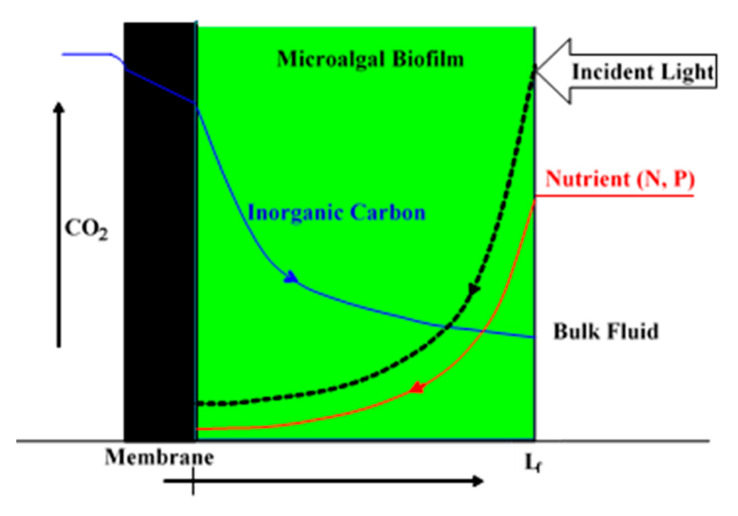
Schematic diagram of the counter-current diffusion and reaction and concentration profiles of inorganic carbon, nutrients (N and P), and light in an MC-MBPBR.

By contrast, biofilm cultivation systems grow microalgae on a solid support rather than freely suspended in liquid medium [1,2,11,12,13]. Microalgal biofilm (MB) cultivation is relatively new and not as broadly and intensely investigated when compared to suspended microalgal cultures [1,2,14,15]. One key advantage of MB is the much higher cell density (up to 100 times that of suspended cell concentrations), which greatly reduces harvesting and dewatering effort, as the MB can simply be scraped and/or vacuumed off the surface and no further concentration is needed [11,12,14,15,16]. However, for commercial applications of an MB system, the questions of economic MB cultivation and harvest, effective CO_2_ and photon delivery and utilization, and optimal energy efficiencies need to be answered. The high cell density in MB needs higher CO_2_ and photon fluxes into MB for growth [17,18], and an optimal MB thickness may exist for maximal CO_2_, nutrients and photon fluxes to the MB, as observed in bacteria membrane aerated biofilm reactors (MABRs) for O_2_ and substrates [19,20,21,22]. Usually, the higher the concentration of CO_2_, the better the suspended microalgae and MB growth, and hence the higher the productivity will be [18,23,24]. The conventional strategies of directly bubbling CO_2_ gas into photobioreactors or open ponds represent a low CO_2_ transfer and utilization efficiency with 50–90% of the CO_2_ escaping back to the atmosphere [25]. Several technologies, such as microbubbles and porous membrane spargers, have been tested to increase CO_2_ transfer efficiency [25]. However, these bubbling CO_2_ delivery technologies seem unable to eliminate the need for surface energy for bubble formation, not to mention the energy consumption associated with the compression and transportation of CO_2_ as well as the low CO_2_ utilization efficiency [25]. Considering the fact that over 50% of the raw material costs in microalgae cultivation are associated with CO_2_, novel CO_2_ delivery and utilization technologies are vital for efficient microalgal production [25]. In recent years, the novel and emerging concept of membrane carbonated microalgal biofilm photobioreactor (MC-MBPBR) technology has been proposed to integrate the advantages of membrane bubbleless molecular CO_2_ transfer (a high CO_2_ transfer rate into an MB with a low energy demand and a high CO_2_ utilization efficiency) and high cell density of MB grown on membrane surfaces for MB cultivation, harvest, and wastewater treatment via a synergistic effect [26,27,28,29,30,31]. As shown in Figure 1, in an MC-MBPBR, the MB is immobilized on the outside of a gas permeable hydrophobic membrane through which CO_2_ molecules are supplied for MB cultivation from the lumen side, while the nutrients (N and P) in wastewater and the photons are transported into the MB from the opposite direction. Lighting from both sides of the biofilm is also feasible, if a transparent gas-permeable membrane is used for both CO_2_ delivery and light penetration to the bottom of the biofilm at the membrane–biofilm interface to overcome the photo limitation of thick biofilm.

This novel and emerging concept of the MC-MBPBR has received much attention in recent years [26,27,28,29,30,31], due to its obvious advantages of significant energy reduction in carbonation and biomass harvesting. In the MC-MBPBR, a hydrophobic membrane is used as the support media for both the microalgal biofilm attachment and molecular CO_2_ delivery into the biofilm for bioreaction. The CO_2_ and the nutrients (N and P) are diffused into the membrane-attached biofilm in a counter-current manner for reaction and thus have a high process and energy efficiency, as compared to that of the conventional biofilm photobioreactors, which use a co-current diffusion. Furthermore, the MC-MBPBR has the potential to achieve 100% CO_2_ utilization and thus significant reduction in carbonation energy. There, however, were only a few experimental studies in the literature [26,27,28,29,30,31] to demonstrate the feasibility of this novel and emerging concept for wastewater treatment. Considering the complexity in the design and operation of MC-MBPBRs, it is highly desirable to use mathematical modelling and simulation techniques to achieve optimization, thus saving cost and time in experimental studies [27].

A membrane aerated biofilm reactor (MABR), which uses a gas-permeable membrane to deliver molecular O_2_ to the membrane-attached bacteria biofilm for biodegradation, is a matured and advanced membrane technology having full-scale applications. Considerable efforts have been made in developing and applying mathematical modelling to guide the design and operation of MABRs [21,32]. Lu et al. [22] summarized the recent advances in mathematical modelling of MABRs. Furthermore, Garg [27] conducted the pioneering work of mathematical modelling of an MC-MBPBR system. However, to the best knowledge of the authors, there is no journal publication on the mathematical modelling of diffusion and reaction of CO_2_ and nutrients (N and P) in a gas-permeable membrane attached to microalgal biofilm. Although there are similarities between MABRs and MC-MBPBRs, the mechanisms of pollutants removal in MABRs and MC-MBPBBRs are different, and thus there are significant differences in the mathematical models. In the MC-MBPBRs, a multiple-factor (CO_2_, N, and P) Monod equation is used, while in the MABRs, a two-factor Monod equation (O_2_ and COD) is often used [21]. Furthermore, light attenuation could be another factor affecting microalgae growth. Thus, the mathematical models for the MC-MBPBRs would be much more complex than those for the MABRs.

The present study aims to fill this gap by developing a set of mathematical equations to describe the diffusion and reaction of CO_2_ and nutrients (N and P) in gas-permeable hydrophobic membrane-attached microalgal biofilm. The impacts of CO_2_ partial pressure, wastewater characteristics (N and P concentrations, and N/P ratios), and biofilm thickness on the biological performance (concentration profiles, and CO_2_, N, and P fluxes into biofilm) of an MC-MBPBR were systematically studied. Furthermore, the proposed mathematical model was validated against experimental results from the literature. It is anticipated that the developed mathematical models will be a powerful tool to guide the design and selection of optimal process parameters for maximum pollutant removal and microalgae productivity.

## 2. Materials and Methods

### 2.1. Basic Governing Equations

The schematic diagram of the MC-MBPBR is shown in Figure 1. The microalgal biofilm is attached on a flat-sheet gas-permeable hydrophobic membrane. The following assumptions are made to derive the governing ordinary differential equations (Equations (1)–(3)) of CO_2_, N, and P diffusion and reactions of CO_2_ and nutrients (N and P) in the biofilm:

(1) That a quasi-steady state of the biofilm thickness can be assumed under tested conditions. That is, the biofilm thickness does not change with time. This is reasonable, considering that the growth rate of biofilm thickness is much smaller than the diffusion and reaction of CO_2_, N, and P in microalgal biofilm;
(2) That there is no limitation on light penetration under tested conditions. Thus, the impact of light intensity is neglected in the present study in order to simplify the models. This could be true for cases of high light intensity illumination, a thin layer of biofilm, pulsed and flashing light, and lighting on both sides (top and bottom) of the biofilm;
(3) That CO_2_, N, and P concentrations are the limiting factors, and the Monod equations can be used to describe the microalgal microbial kinetics;
(4) That CO_2_ is the only inorganic carbon source for microalgal biofilm development;
(5) That pH effects and CO_2_ speciation (CO_2_, HCO_3_^−1^, CO_3_^2−^) are excluded;
(6) That the MC-MBPBR is operated at ambient temperature (around 25 °C).

In the membrane-attached microalgal biofilm, the molecular diffusion of CO_2_, N, and P is governed by Fick’s law; the consumption (reaction) of these molecules and ions occurs simultaneously. The microbial kinetics of microalgae are described by the multifactor Monod equation [33]. Thus, the quasi-steady-state ordinary differential equations governing the diffusion and reaction of CO_2_, N, and P in a flat-sheet gas-permeable hydrophobic membrane-attached microalgal biofilm, based on the concept of mass balances, are as follows:

(1) $$D_{{CO}_{2}-eff}\frac{d^{2}S_{{CO}_{2}}}{dx^{2}}-\;\mu_{max-m}\frac{S_{{CO}_{2}}}{K_{{CO}_{2}}+S_{{CO}_{2}}}\frac{S_{N}}{K_{N}+\;S_{N}}\frac{S_{P}}{K_{P}+\;S_{P}}Y_{M-{CO}_{2}}X_{m}\;=0,$$

(2) $$D_{N-eff}\frac{d^{2}S_{N}}{dx^{2}}-\mu_{max-m}\frac{S_{{CO}_{2}}}{K_{{CO}_{2}}+S_{{CO}_{2}}}\frac{S_{N}}{K_{N}+S_{N}}\frac{S_{P}}{K_{P}+S_{P}}\frac{X_{m}}{Y_{M-N}}=0,$$

(3) $$D_{P-eff}\frac{d^{2}S_{P}}{dx^{2}}-\mu_{max-m}\frac{S_{{CO}_{2}}}{K_{{CO}_{2}}+S_{{CO}_{2}}}\frac{S_{N}}{K_{N}+S_{N}}\frac{S_{P}}{K_{P}+S_{P}}\frac{X_{m}}{Y_{M-P}}=0,$$

where *D*_CO2*-eff*_, *D_N-eff_*, and *D_P-eff_* are the effective diffusivity of CO_2_, N, and P in the biofilm, respectively, m^2^/s; *x* is the distance from the membrane–biofilm interface (where *x* = 0) in the biofilm thickness direction, m; *S_CO_*_2_, *S_N_*, and *S_P_* are the concentration of CO_2_, N, and P in the biofilm, respectively, kg/m^3^; *µ_max-m_* is the maximum specific growth rate of the microalgae, 1/s; *K_CO_*_2_, *K_N_*, and *K_P_* are the Monod half-saturation constants for CO_2_, N, and P, respectively, g/m^3^; *X_m_* is the microalgal concentration in the biofilm, g(dry biomass)/m^3^; *Y_M-CO_*_2_ is the microalgal CO_2_ consumption coefficient, g CO_2_ consumed/g biofilm produced; and finally, *Y_M-N_*, and *Y_M__-P_* are the microalgal biofilm yields, based on N and P consumptions, with the units of g biofilm/g N consumed and g biofilm/g P consumed, respectively.

### 2.2. Boundary Conditions

At the membrane and biofilm interface (*x* = 0), the membrane is impermeable to nutrients (N and P) and water. Similar to the impermeable conditions for bacteria MABR models [19,20,21,22], the boundary conditions for the nutrients (N and P) at *x* = 0 are as follows:

(4) $$x=0,\;\frac{dS_{N}}{dx}{|}_{x=0}=0,\;\frac{dS_{P}}{dx}{|}_{x=0}=0,$$

On the other hand, the membrane is permeable for CO_2_ molecules and delivers molecular CO_2_ to the microalgal biofilm. Similar to the condition of O_2_ transferring across the membrane in bacteria MABRs [19,20,21,22], the flux of CO_2_ transferred across the membrane should equal that diffused into the biofilm at *x* = 0 by Fick’s law:

(5) $$x=0,\;D_{{CO}_{2}-eff}\frac{dS_{{CO}_{2}}}{dx}{|}_{x=0}=\;-\;\frac{HP_{m}}{L}\left(\frac{44P_{0}}{H}-S_{{CO}_{2}-0}{|}_{x=0}\right),$$

where *H* is the Henry’s law constant of CO_2_, m^3^·atm/mol CO_2_; *P_m_* is the permeability coefficient of CO_2_ across the membrane, mol CO_2_/atm·s·m; *P_o_* is the partial pressure of CO_2_ in the gas flowing through the lumen side of the membrane, atm; and finally, *L* is the thickness of the membrane, m.

At the biofilm and bulk water interface (*x* = *L_f_*, the biofilm thickness), assuming S_CO2_ = 0 mg/L in the bulk phase,

(6) $$x=L_{f},\;D_{{CO}_{2}-eff}\frac{dS_{{CO}_{2}}}{dx}{|}_{x=L_{f}}=-D_{{CO}_{2}-H_{2}O}\frac{S_{{CO}_{2}}{|}_{x=L_{f}}-0}{L_{b}},$$

(7) $${x=L_{f},\;D}_{N-eff}\frac{dS_{N}}{dx}{|}_{x=L_{f}}=D_{N-H_{2}O}\frac{S_{N-b}-S_{N}{|}_{x=L_{f}}}{L_{b}},$$

(8) $${x=L_{f},\;D}_{P-eff}\frac{dS_{P}}{dx}{|}_{x=L_{f}}=D_{P-H_{2}O}\frac{S_{P-b}-S_{P}{|}_{x=L_{f}}}{L_{b}},$$

where *D_CO_*_2-*H*2*O*_, *D*_*N*-*H*__2*O*_, and *D*_*P*-*H*__2*O*_ are the diffusivity of CO_2_, N, and P in water, respectively, m^2^/s; *S*_*N*-*b*_ and *S_P-b_* are the concentration of N and P in the bulk phase of wastewater, respectively, g/m^3^; and *L*_*b*_ is the thickness of the stagnant boundary layer of water on the surface of the microalgal biofilm, m.

### 2.3. Numerical Methods

To simplify the computations, the linear finite-difference method was used to establish the tri-diagonal Jacob matrix, and iteration was conducted to find the converged CO_2_, N, and P concentration profiles and the fluxes of CO_2_ (g CO_2_/m^2^·s) and nutrients (N and P) (g N/m^2^·s or g P/m^2^·s) to the microalgal biofilm. The impacts of CO_2_ partial pressure, influent NO_3_^−^-N and HPO_4_^2−^-P concentrations, and biofilm thickness on the biological performance of the MC-MBPBR were investigated. MATLAB^®^ software (2024a version) was employed to iteratively solve the linearized tri-diagonal Jacob matrix until convergence and to perform case studies, graphing, and relevant analyses.

**Figure 2:**
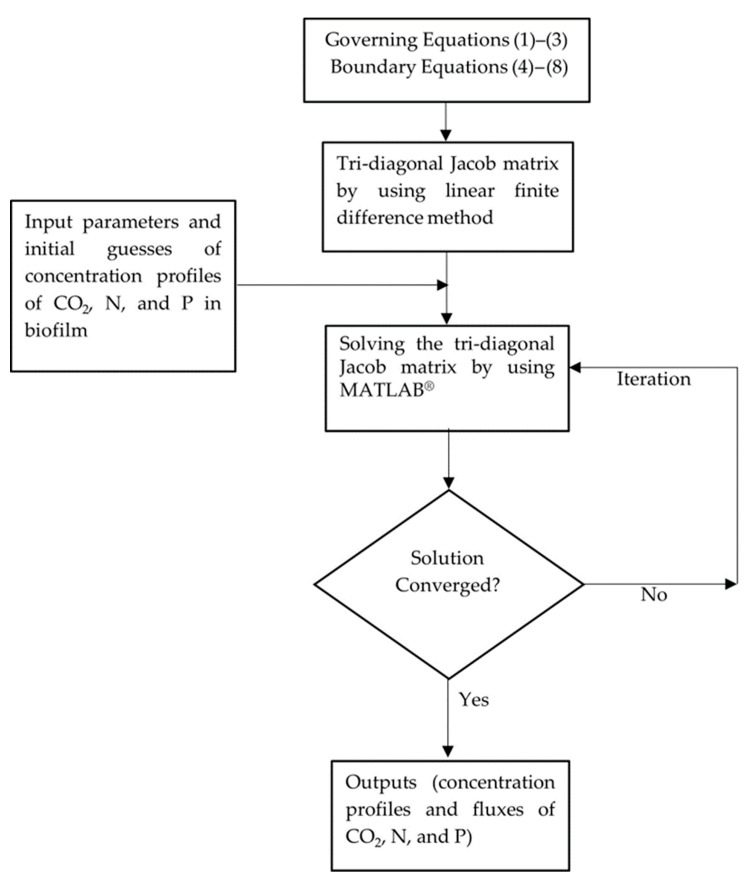
Schematic diagram of modelling flowchart.

A schematic of the modelling workflow is presented in Figure 2.

### 2.4. Values of Parameters

The values of the parameters involved in the governing equations and boundary conditions, including the microbial kinetic constants and stoichiometric constants, are extracted from the literature directly, estimated based on experimental results from the literature, or assumed based on the typical range of these parameter values in the literature. The values of the parameters (microbial kinetic constants and stoichiometric coefficients, diffusivities, influent concentrations of N and P, and the CO_2_ concentration in the gas phase) used in the studies are summarized in Table 1.

### 2.5. Validation of Mathematical Models

The accuracy of the prediction of CO_2_, N, and P fluxes into the membrane-attached microalgal biofilm was verified by using the experimental results [28] from the literature.

The following experimental conditions were used by Guo et al. [28] to obtain the experimental results used for the validation of the mathematical models developed here: the microalgae strain was a freshwater *Scenedesmus obliquus* FACHB-13 (*S. obliquus*) from the Freshwater Algae Culture Collection of Hydrobiology, Chinese Academy of Science, China. Blue-Green medium (BG11) (1.5 g NaNO_3_ /L; 0.04 g K_2_HPO_4_/L; and other nutrients (salts) and trace metal ions) was used to develop microalgae for the MC-MBPBR experiment. Polytetrafluoroethylene (PTFE) membrane coated on a stainless-steel mesh was used as the gas-permeable hydrophobic membrane in the MC-MBPBR to deliver CO_2_ for microalgal biofilm development for wastewater treatment. The continuous MC-MBPBR system (open loop) was composed of a liquid chamber (150 mm × 60 mm × 6 mm) on the top and a gas chamber (150 mm × 60 mm × 4 mm) on the bottom. The liquid and gas flow rates tested were 1–4 mL/h and 1–7 mL/min, respectively. The gaseous CO_2_ concentration entering the gas chamber was 10% (*v*/*v*). Experimental temperature was controlled at 25 ± 1 °C.

### 2.6. Sensitivity Analysis

Sensitivity analysis of the mathematical models was conducted to investigate the importance of some key input parameters—such as the effective diffusivities of CO_2_, N, and P (*D*_CO2-*eff*_, *D_N-eff_*, and *D_P-eff_*) in the biofilm, the maximum specific growth rate of microalgae (*µ_max-m_*), the biofilm density (*X_m_*, microalgal concentration in biofilm), in controlling the CO_2_, N, and P fluxes into the biofilm. A change of ±10% of the values of these input parameters was tested to evaluate their impacts on the CO_2_, N, and P fluxes. The relative errors of these fluxes between the base line and the new values (after ±10% change) of these parameters were calculated to rank the relative importance of these parameters in controlling these fluxes.

## 3. Results and Discussion

**Figure 3:**
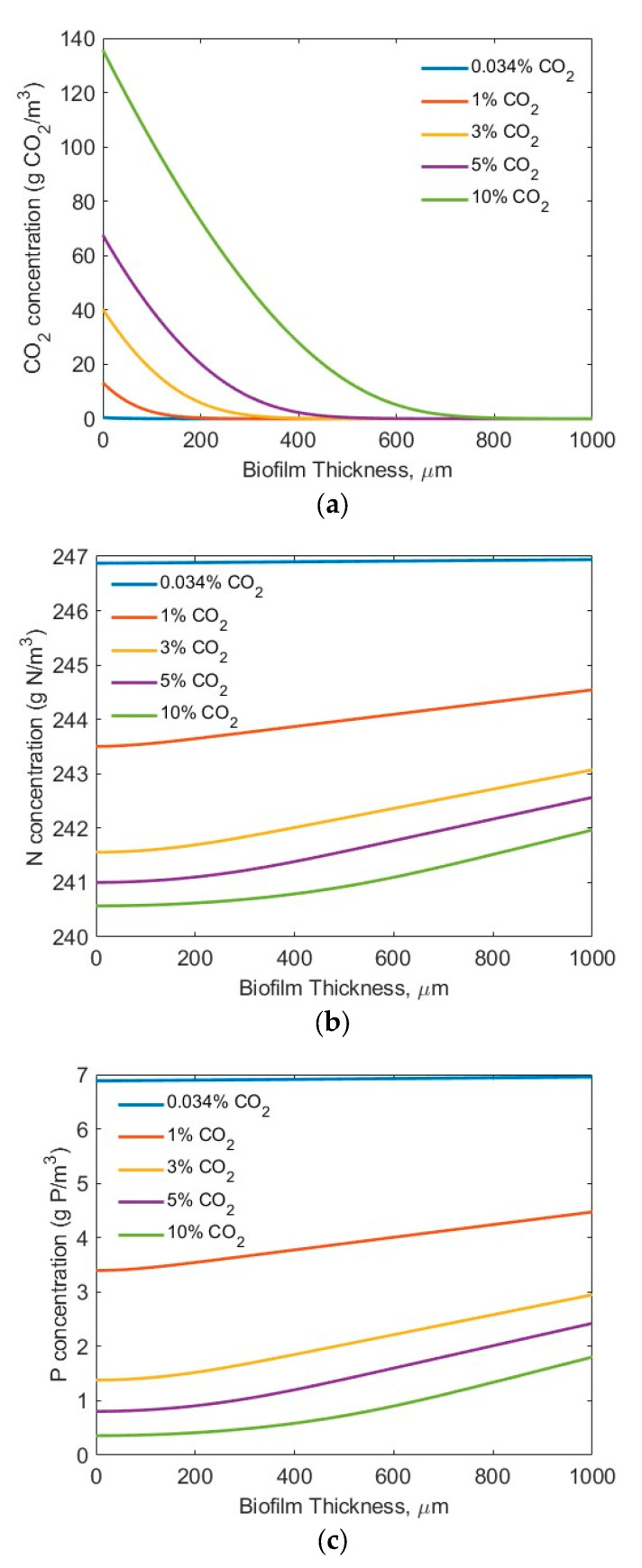
Modelling results of the concentration profiles (influents *S_N-b_* = 247.1 mg N/L; *S_P-b_* = 7.12 mg P/L; assuming *L_f_* = 1000 µm): (**a**) CO_2_; (**b**) N; and (**c**) P in biofilm.

**Figure 4:**
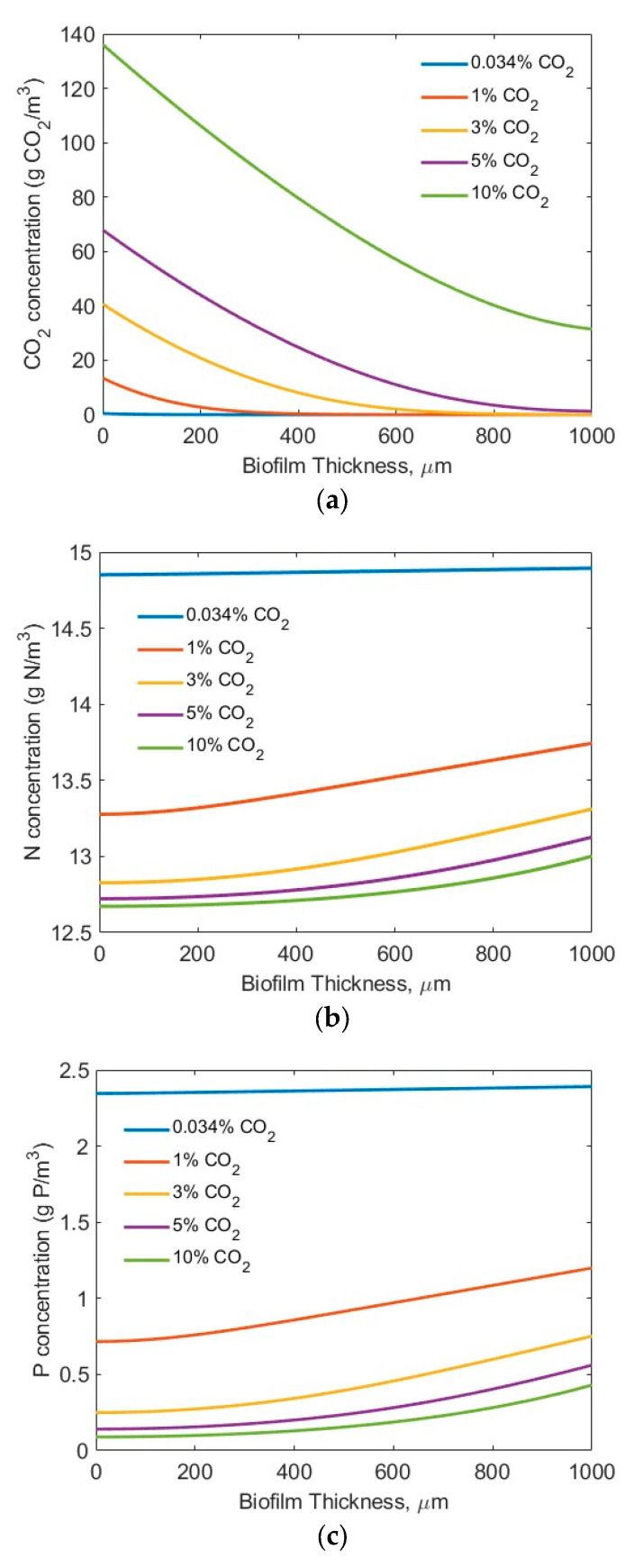
Modelling results of the concentration profiles (influents *S_N-b_* = 15 mg N/L; *S_P-b_* = 2.5 mg P/L; assuming *L_f_* = 1000 µm): (**a**) CO_2_; (**b**) N; and (**c**) P in biofilm.

**Figure 5:**
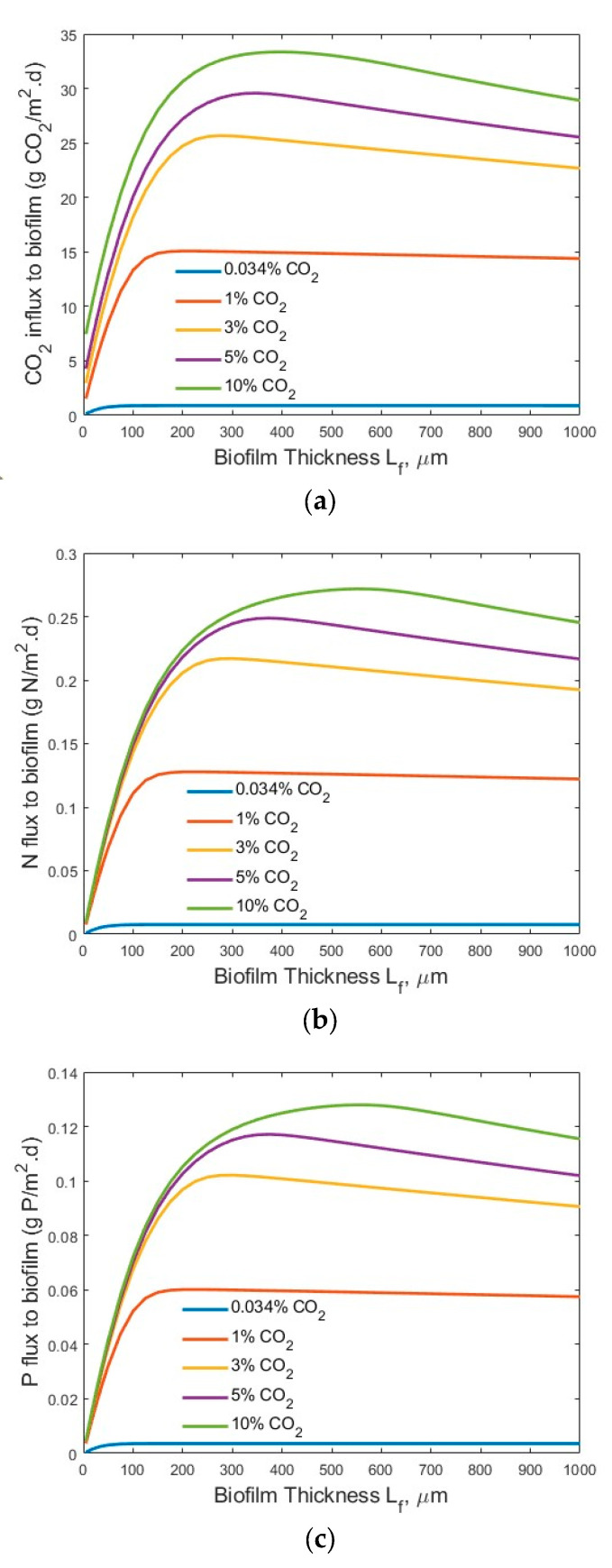
Modelling results of the flux profiles (influents *S_N-b_* = 247.1 mg N/L; *S_P-b_* = 7.12 mg P/L; *L_f_* = 0–1000 µm): (**a**) CO_2;_ (**b**) N; and (**c**) P into biofilm.

For the modelling studies, five CO_2_ concentrations (0.034% (air), 1%, 3%, 5%, and 10%) were assumed. No other, higher CO_2_ concentration was tested, considering the potential inhibiting effect of a high CO_2_ concentration on microbial biofilm growth [44]. Two sets of nutrient (N and P) conditions were tested (high-strength industrial wastewater (*S_N-b_* = 247.1 mg N/L; *S_P-b_* = 7.12 mg P/L) [28] and low-strength wastewater (*S_N-b_* = 15 mg N/L; *S_P-b_* = 2.5 mg/L) similar to effluents from secondary municipal wastewater treatment). Also assumed was the biofilm thickness *L_f_* = 0–1000 µm, based on the penetration limitations of CO_2_, N, P, and light. Figure 3 and Figure 4 show the concentration profiles of CO_2_, N, and P in the biofilm. Figure 4 and Figure 5 display the impact of biofilm thickness on CO_2_, N, and P fluxes into the biofilm.

### 3.1. Concentration Profiles of CO_2_, Total Nitrogen (TN), and Total Phosphorus (TP) into the Biofilm

The modelling results suggest that the penetration distance of the CO_2_ (Figure 3a and Figure 4a) increased with an increase in the bulk gas phase CO_2_ concentration. Under the high-strength industrial wastewater conditions (influents *S_N-b_* = 247.1 mg N/L; *S_P_-_b_* = 7.12 mg P/L), even at the highest tested CO_2_ concentration of 10%, the penetration distance of the CO_2_ was approximately 700 µm, and much smaller biofilm penetration distance was observed at lower bulk gas phase CO_2_ concentration (Figure 3a). On the other hand, under the low-strength wastewater conditions (*S_N-b_* = 15 mg N/L; *S_P-b_* = 2.5 mg P/L), a penetration of CO_2_ into the wastewater stream at CO_2_ concentrations of 3% and 10% was observed (Figure 4a). Not all the CO_2_ transferred across the membrane into the biofilm was utilized for bioreaction. These results suggest that the utilization of the CO_2_ would change in terms of the biofilm thickness and influent nutrient conditions. Thus, an optimal gas phase CO_2_ concentration could be identified for practical application, based on the influent nutrients’ conditions and the biofilm thickness.

Figure 3b and Figure 4b suggest that the TN penetrated through the entire biofilm to the membrane–biofilm interface (*x* = 0) and that TN is available for bioreaction across the entire spread (thickness-wise) of the biofilm. However, the penetration distance of the TP was limited (Figure 3c and Figure 4c) under the tested conditions. A very low TP concentration (<0.5 mg P/L) at the membrane–biofilm interface (*x* = 0) was observed even under the conditions of high TP concentration (*S_P-b_* = 7.12 mg P/L) and high bulk gas phase CO_2_ concentration (10%). The limitation of TP (Figure 3c) was worsened under the low-strength wastewater conditions (*S_P-b_* = 2.5 mg P/L). Extremely low TP concentrations of less than approximately 0.25, 0.15, and 0.1 mg P/L at the membrane–biofilm interface (*x* = 0, Figure 4c) were observed at the bulk gas phase CO_2_ concentrations of 3%, 5%, and 10%, respectively. These results suggest that the CO_2_, TN, and TP could all be the potential limiting factors for bioreaction, depending on the tested process conditions. A further increase of the bulk gas phase CO_2_ concentration would increase its penetration distance; however, there is a limitation of the highest CO_2_ concentration that would not inhibit microalgae growth [43]. In the meantime, the entire spread (thickness) of the biofilm was not used for reaction. A significant increase in biofilm thickness only increased the mass transfer resistance for diffusion of the CO_2_, TN, and TP, but a large portion of the biofilm did not participate in the bioreaction, due to a lack of CO_2_, TN, and/or TP molecules or ions in that portion of the biofilm. This suggests the importance of controlling the biofilm thickness.

### 3.2. Transfer Fluxes of CO_2_, TN, and TP into the Biofilm

**Figure 6:**
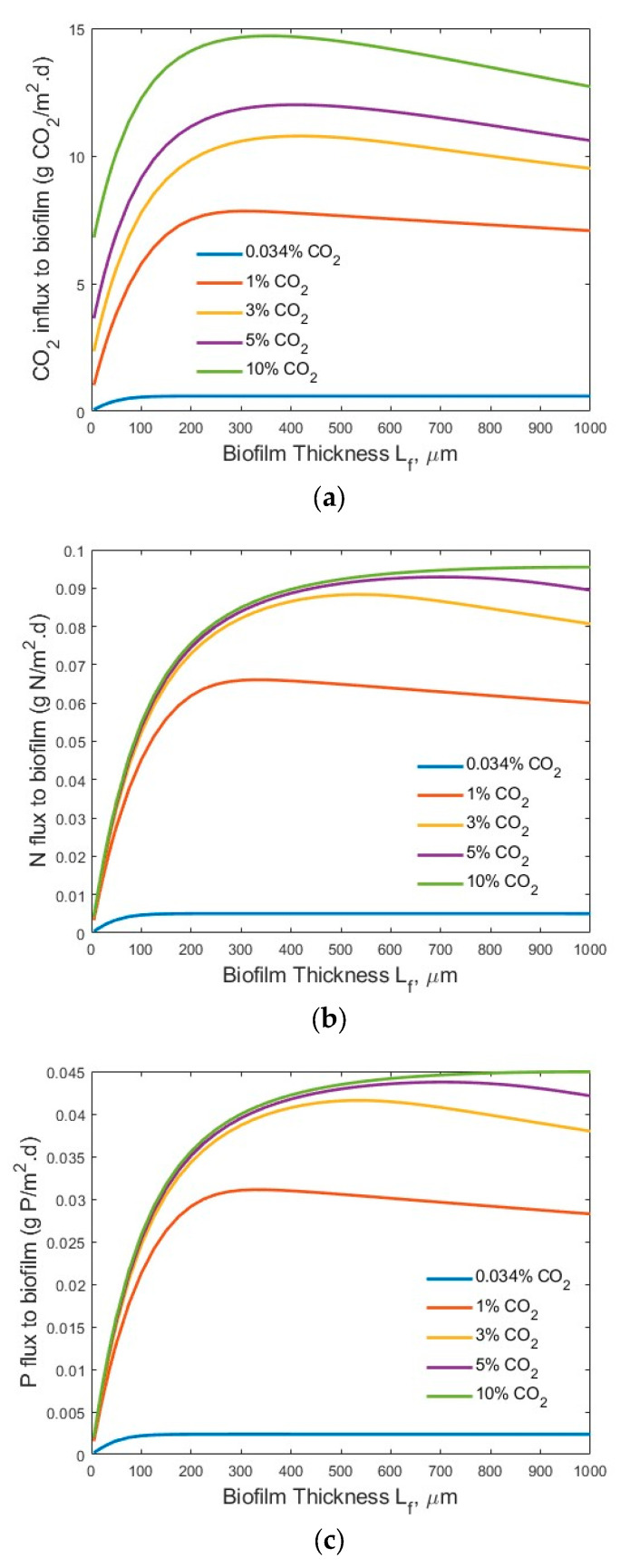
Modelling results of the flux profiles (influents *S_N-b_* = 15 mg N/L; *S_P-b_* = 2.5 mg P/L; *L_f_* = 0–1000 µm): (**a**) CO_2_; (**b**) N; and (**c**) P into biofilm.

Figure 5 and Figure 6 show the transfer flux profiles of CO_2_, TN, and TP into the biofilm under the tested process conditions. A much higher CO_2_ flux (g CO_2_/m^2^·d) into the biofilm (Figure 5a) was observed under the high-strength industrial wastewater conditions, as compared to that under the low-strength wastewater conditions (Figure 6a). This is not surprising, as an increase in the wastewater strength (nutrient levels) would increase the transfer of nutrients (TN and TP) into the biofilm for bioreaction, as shown in Figure 5b and Figure 6b, and Figure 5c and Figure 6c, respectively, and thus consume more CO_2_ for microalgae biofilm growth and drive the increase of CO_2_ flux into the biofilm. Similar trends were observed for the TN fluxes (Figure 5b vs. Figure 6b) and TP fluxes (Figure 5c vs. Figure 6c) under the high-strength industrial wastewater conditions. An increase in influent TN and/or TP concentration would increase the driving force of mass transfer of TN and TP from the bulk wastewater stream to the biofilm for bioreaction.

It is interesting to observe that an optimal biofilm thickness existed (Figure 5 and Figure 6) under each set of tested process conditions. The results, shown in Figure 4 and Figure 5, suggest that the concentration of CO_2_, TN, and TP concentration had a significant impact on the optimal biofilm thickness and the process efficiency (fluxes into the biofilm). The optimal biofilm thickness was found to be approximately 220, 220, 275, 350, and 400 µm for maximum CO_2_ flux, and approximately 225, 225, 300, 350, and 500 µm for maximum N and P fluxes, under the bulk gas phase CO_2_ concentrations of 0.034%, 1%, 3%, 5%, and 10%, respectively, and high-strength industrial wastewater conditions. The slight differences of optimal biofilm thicknesses between maximum CO_2_ flux and maximum TN and TP fluxes are because CO_2_, TN, and TP are transferred into the defined biofilm zone (or grid cells) for bioreaction in a counter-current diffusion manner. Therefore, CO_2_ and TN/TP reach maximum flux at different positions in the biofilm (from the membrane–biofilm interface to the biofilm–bulk wastewater stream interface).

It is noted that under the low-strength wastewater conditions, an optimal biofilm thickness existed for both a maximum CO_2_ (Figure 6a) and maximum TN (Figure 6b) and TP (Figure 6c) fluxes into the biofilm at the bulk gas phase CO_2_ concentrations of 0.034%, 1%, 3%, and 5%, respectively. However, no optimal biofilm thickness for a maximum TN (Figure 6b) and TP (Figure 6c) was reached under the tested biofilm thickness L_f_ (1000 µm) at the bulk gas phase CO_2_ concentration of 10%. This was because a portion of the CO_2_ penetrated through the entire biofilm and transferred into the bulk wastewater stream at the bulk gas phase CO_2_ concentration of 10%. In other words, not all the CO_2_ transferred through the membrane into the biofilm participated in the bioreaction. Further increase in the biofilm thickness (>1000 µm) would reduce the amount of CO_2_ that penetrated through the entire biofilm and into the bulk wastewater stream and increase the amount of CO_2_ for bioreaction, which needs more TN and TP simultaneously, and increase the TN and TP fluxes. Thus, the optimal biofilm thickness for a maximum TN and TP flux into the biofilm would be larger than 1000 µm, and it would be achieved when the biofilm thickness was great enough for all the CO_2_ transferred through the membrane to be consumed in the biofilm for bioreaction; that is, no CO_2_ was released into the bulk wastewater stream at a bulk gas phase CO_2_ concentration of 10%.

The existence of an optimal microalgal biofilm thickness (*L_f_*) for maximum CO_2_, TN, and TP fluxes into the biofilm under different experimental conditions was clearly demonstrated by this modelling study, as shown in Figure 5 and Figure 6. This finding is consistent with the observations of the bacteria membrane aerated biofilm reactor (MABR) in terms of the optimal bacteria biofilm thickness for O_2_ and chemical oxygen demand (COD) into the bacteria biofilm [19,20,21,22]. The existence of an optimal biofilm thickness for maximum fluxes could be explained by the facts that are related to the increased mass transfer resistance of a thick biofilm as well as the gas penetration and low biofilm biomass of a thin biofilm. A thin biofilm, which occurs at the beginning of the operation of an MC-MBPBR, would lead to a potential penetration of the CO_2_ molecules through the biofilm into the bulk liquid phase and limited biofilm biomass for biodegradation, thus limiting the N and P fluxes into the biofilm. On the other hand, a thick biofilm would significantly increase the mass transfer resistance of the CO_2_, N, and P in the biofilm and thus reduce the fluxes of CO_2_, N, and P into the biofilm. As shown in Figure 4 (secondary effluent treatment), at a biofilm thickness of 700 µm, the CO_2_ concentration is near zero, while at the membrane–biofilm interface (*x* = 0), the P concentration is <0.5 mg/L. Consequently, based on the multiple-factor Monod equation, the microalgae cell synthesis growth rate would be very small at both the biofilm–water and membrane–biofilm interfaces and lead to reduced CO_2_, N, and P fluxes into the biofilm. Thus, strategies such as intermittent aeration for biofilm thickness control should be developed in the design and operation of the novel and emerging MC-MBPBR for controlling the optimal biofilm thickness. Another technique that can be used for biofilm thickness control in MC-MBPBRs is ultrasonic biofilm detachment [44]. Acoustic cavitation has been proved to be an effective method for biofilm detachment and thickness control in MABRs [45]. Furthermore, the identified optimal microalgal biofilm thickness (220–500 µm) is in the range of typical light penetration limitation (120–250 µm) [13,18,46,47,48] and nutrient (N and P) penetration limitation (150–355 µm) [48,49] in microalgal biofilm, under some experimental conditions. To improve the light and nutrient (N and P) penetration distances, light intensity and nutrient (N and P) concentrations can be increased. Under a high light intensity, the impact of light intensity on the microalgal biofilm growth might be minimized and thus the light intensity might not be a limiting factor. To overcome the light penetration limitation, lighting on both sides of the microalgal biofilm might also be considered. The use of a transparent hydrophobic membrane for molecular CO_2_ delivery and biofilm attachment can achieve lighting on both sides of the membrane-attached microalgal biofilm. Whenever possible, natural light and environmentally friendly renewable energy should be used for light generation to minimize the energy cost of lighting in the photobioreactor.

### 3.3. Sensitivity Analysis of the Impacts of Critical Parameters

**Figure 7:**
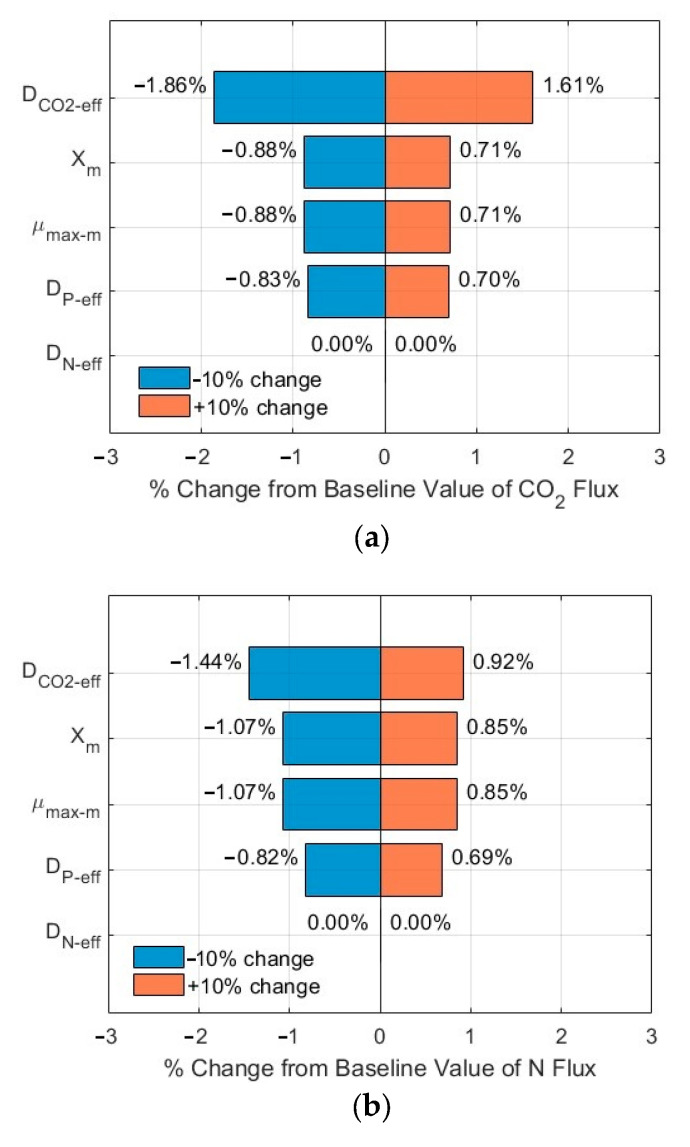

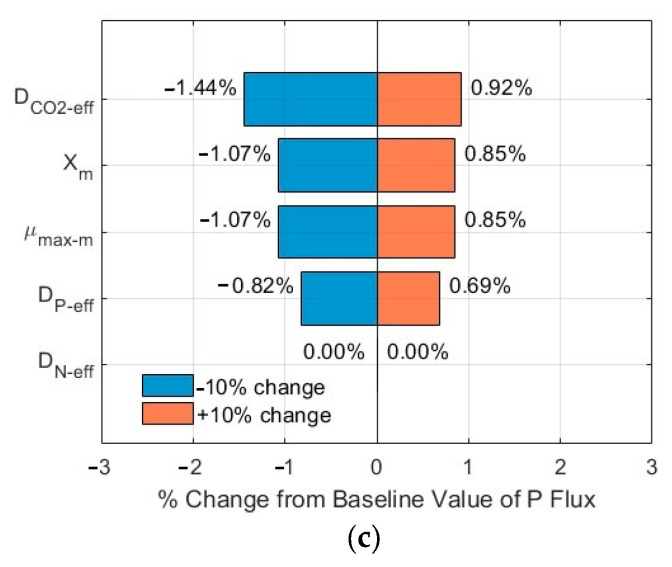
Sensitivity analysis results of fluxes (influents *S_N-b_* = 247.1 mg N/L; *S_P-b_* = 7.12 mg P/L; *L_f_* = 625 µm): (**a**) CO_2_; (**b**) N; and (**c**) P in biofilm.

**Figure 8:**
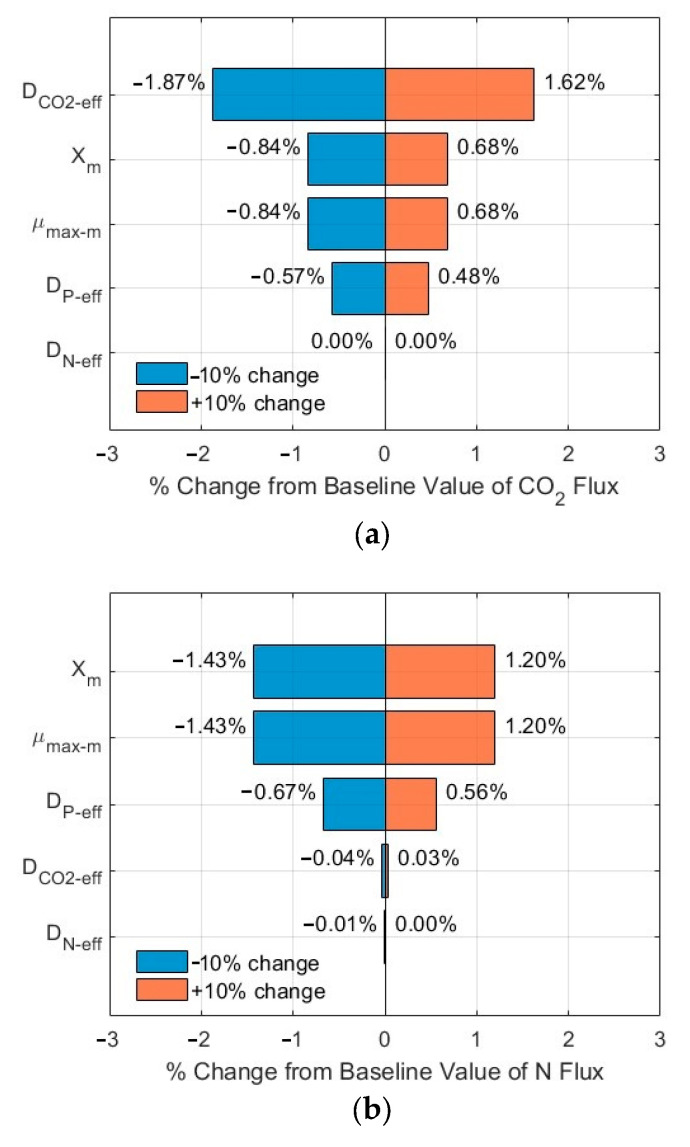

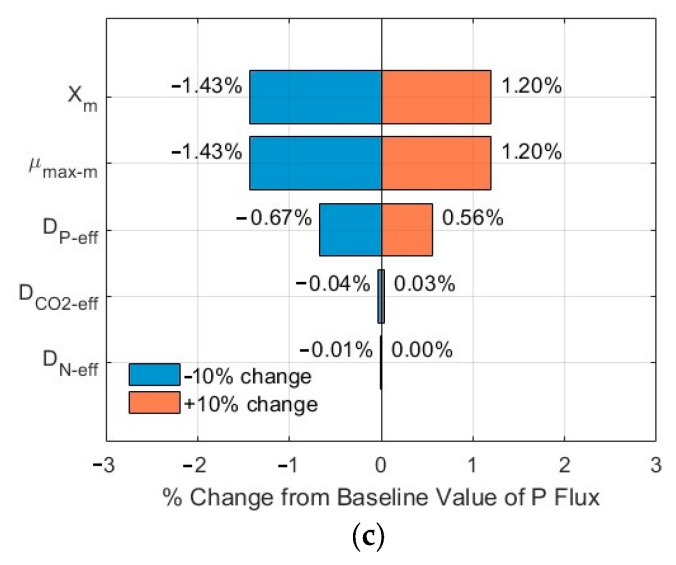
Sensitivity analysis results of fluxes (influents *S_N-b_* = 15 mg N/L; *S_P-b_* = 2.5 mg P/L; *L_f_* = 625 µm): (**a**) CO_2_; (**b**) N; and (**c**) P in biofilm.

The tornado plots shown in Figure 7a–c and Figure 8a–c show the ranking of importance of some critical input parameters (*D*_CO2*-eff*_, *D_N-eff_*, *D_P-eff_*, *µ_max-m_*, *X_m_*) affecting the CO_2_, N, and P fluxes into the biofilm. As compared to the fluxes from the base line, a change of ±10% in the values of these critical parameters, with *S_N-b_* = 247.1 mg N/L*, S_P-b_* = 7.12 mg P/L, and *L_f_* = 625 µm, resulted in a relative error in the range of −1.86–1.61%, −1.44–0.92%, and −1.44–0.92% for the CO_2_, N, and P fluxes, respectively. The importance ranking in the order from high to low is *D*_CO2-*eff*_ > *X_m_* and *µ_max-m_* > *D_P-eff_* > *D_N-eff_* for the CO_2_ flux (Figure 7a); the same ranking holds for the N flux (Figure 7b) and for the P flux (Figure 7c). On the other hand, with *S_N-b_* = 15 mg N/L, *S_P-b_* = 2.5 mg P/L, and *L_f_* = 625 µm, the relative errors are in the range of −1.87–1.62%, −1.43–1.20%, and −1.43–1.20% for the CO_2_, N, and P fluxes, respectively. The importance ranking in the order from high to low is *D*_CO2-*eff*_ > *X_m_* and *µ_max-m_* > *D_P-eff_* > *D_N-eff_* for the CO_2_ flux (Figure 8a); it is *X_m_* and *µ_max-m_* > *D_P-eff_* > *D*_CO2-*eff*_ > *D_N-eff_* for the N flux (Figure 8b) and for P flux (Figure 8c).

The sensitivity analysis shows that the modelling results are relatively stable, with a maximum relative error of 1.87% under a change of ±10% of these critical parameters. Furthermore, the order of the importance of these critical parameters might vary, depending on the wastewater characteristics (N and P levels and ratios). The minimum impact of D_N-eff_ might be related to the high concentration of TN in the biofilm, as shown in Figure 3b and Figure 4b, which would suggest TN might not be a limiting agent for microalgae biofilm growth and nutrient removal. The same magnitude of impacts of *µ_max-m_* and *Xm* on the relative errors might be related to the fact that these two parameters are included in Equations (1)–(3) only and as a product *µ_max-m_* × *Xm*, thus having the same impact.

### 3.4. Validation of the Mathematical Models with Experimental Results from the Literature

As the MC-MBPBR is an emerging and innovative concept for wastewater treatment, there are limited experimental studies of feasibility tests reported in the literature [26,27,28,29,30,31]. The accuracy of the mathematical models proposed in this study was validated by using the experimental results of Guo et al. (2019) [28], one of the few experimental studies available from the literature. Table 2 summarizes the comparison of CO_2_ and TN fluxes into the biofilm between the modelling results and experimental results from the literature (detailed calculations of the experimental CO_2_ and N fluxes into the microalgal biofilm from Guo et al. (2019) [28] are provided in the Supplementary Materials). The modelling results of CO_2_ (32.11 g CO_2_/m^2^·d) and N (0.178 g N/m^2^·d) fluxes into the biofilm were in good agreement with the experimental results (CO_2_: 34.58–62.48 g CO_2_/m^2^·d with an average of 53.4 g CO_2_/m^2^·d; N: 0.148–0.214 g N/m^2^·d with an average of 0.180 g N/m^2^·d). It should be noted that the above experimental results (CO_2_: 34.58–62.48 g CO_2_/m^2^·d; N: 0.148–0.214 g N/m^2^·d) were not first-hand data; instead, they were calculated, based on the experimental results from Guo et al. (2019) [28], under similar experimental conditions. The relative error for the CO_2_ flux into the biofilm was in the range of 7.14% to 48.6%, while the relative error for the TN flux into the biofilm was in the range of −20.3% to 16.8%. From Table 2, it is noted that an increase in the gas flow rate (1 to 5 mL/min) in the gas chamber resulted in an increase in the relative experimental errors for the CO_2_ flux into the biofilm. This could be due to the fact that an increase in the gas flow rate would cause a more turbulent flow in the gas chamber which would disturb the PTFE membrane and biofilm structure (likely resulting in biofilm detachment). Considering the nature of the comparison of process efficiencies, the modelling results were deemed in good agreement with the experimental results. This is due to two aspects. Experimentally speaking, the results were affected by factors such as errors associated with gas and liquid flow rate control and measurement, CO_2_ and TN measurements, and potential system leakage. On the other hand, mathematical modelling and computation were affected by the accuracy of the microbial kinetic constants and stoichiometric coefficients and the assumptions made in modelling. From the modelling perspective, the CO_2_ flux was found at the low end of the experimental CO_2_ flux into the biofilm. The reason could be that some CO_2_ diffused through the membrane escaped from the biofilm into the bulk wastewater in the experimental study, as mentioned by the authors [27,28]. This could likely be caused by potential minor system and/or membrane leaking into the bulk wastewater, CO_2_ penetration of an uneven thin-layer biofilm, and CO_2_ micro-bubbles being transferred from a few large pores of the PTFE membranes through the voids of biofilm into the bulk wastewater stream. The modelling results of the TN flux (0.178 g/m^2^·d) into the biofilm are close to the experimental results (0.153–0.214 g N/m^2^·d) of the TN flux into the biofilm. The computed CO_2_ and TN fluxes into the biofilm seem reasonable and accurate for bioreaction and microalgae growth. Furthermore, the accuracy of the mathematical modelling results can be further improved by using calibrated microbial kinetic constants and stoichiometric coefficients from the same experimental conditions.

### 3.5. Limitations of This Study

Since this was a modelling study, assumptions were made to establish the mathematical models. Thus, there are limitations in this study. For example, for thick microalgal biofilm, a light shading effect should be considered and included in these models. To consider light attenuation in microalgal biofilm, the simplest model, the Lambert–Beer model (I = I_o_e^−α.L^, where I and I_o_ are the local and incident light intensity, respectively, µmol/m^2^·s; α is the biofilm extinction coefficient, 1/m; L is the biofilm thickness (=*L_f_* − x), m) [50], can be integrated into the multifactor Monod equation in Equations (1)–(3) by multiplying the term of I/(K_I_ + I) (where K_I_ is the half-maximum-rate light irradiance, µmol/m^2^·s). Furthermore, temperature is another important factor; the impact of temperature on the mathematical modelling can be incorporated into the estimation of diffusivities of D_CO2-eff_, D_N-eff_, D_P_-_eff_, and microbial kinetic equations [22,33]. Moreover, CO_2_ dissolved in water would dissociate into HCO_3_^−^ and CO_3_^2−^ and eventually establish an equilibrium with these ions. This would affect the pH of the solution. Peng et al. [51] found that the pH and HCO_3_^−^ concentration had a significant impact on *Neochloris oleoabundans* growth. At a pH of 7.5, an increase in the HCO_3_^−^ concentration (0–160 mM) resulted in a decrease in cell maximum specific growth rate (µ_max-m_) and cell productivity [51]. An increase in pH (7.5–9.5) improved microalgae cell productivity [51]. At a pH range of 7.5–9.5, HCO_3_^−^ is the dominant dissolved inorganic carbon (DIC) species for CO_2_ dissociation [39,51]. Furthermore, for the same amount of DIC produced by CO_2_ gas absorption and NaHCO_3_ dissolution, respectively, the solution pH would be different (lower for CO_2_ absorption) [52]; thus, different CO_2_ speciation would have an impact on microalgae cell yield. By using a CO_2_ delivery strategy of combing gas CO_2_ sparging and NaHCO_3_ dosing, the pH of the cultivation medium can be stabilized and thus the impact of pH change would be minimized [52]. Moreover, the diffusivity of different CO_2_ speciation in the aqueous phase has an order from high to low: D_CO2_ > D_HCO3−_ > D_CO3 2-_ [27]. Although the total inorganic carbon is the same (no matter whether CO_2_ dissociation and equilibrium is considered or not), CO_2_ dissociation and equilibrium would lead to a change of the fraction of CO_2_, HCO_3_^−^, and CO_3_^2−^ in the aqueous phase; consequently, the assumption of CO_2_ as the only inorganic carbon source (by neglecting its dissociation and equilibrium) might overestimate the CO_2_ in the biofilm, as demonstrated in the sensitivity analysis of D_CO2-eff_. However, as a first step towards ultimately optimizing the design and operation of the novel MC-MBPBR, the feasibility of using simplified mathematical models to describe the diffusion and reaction of gaseous CO_2_ and aqueous nutrients (N and P) was successfully achieved. Future modelling studies should incorporate the light intensity factor and the dissociation and equilibrium of CO_2(aq)_, HCO_3_^−^_(aq)_, and CO_3_^2−^_(aq)_ into the mathematical model to provide more comprehensive and in-depth insights into the operation and design of an MC-MBPBR. Moreover, future model development should consider dynamic mathematical models rather than the quasi-steady-state models developed here. A dynamic mathematical model of mass balances of the microalgal biofilm would include not only the biofilm thickness growth with time but also a biofilm detachment term, which is affected by hydrodynamic conditions of both the liquid chamber and gas chamber. An increase in gas flow rate would cause vibration of the membranes and eventually biofilm detachment, while an increase in liquid flow velocity would increase shear stress and thus biofilm detachment. The approach used by Pechaud et al. [53] for estimation of the biofilm growth and detachment terms can be incorporated into microalgal biofilm mass balance of the MC-MBPBR systems in future model development.

The values of the microbial kinetic parameters and stoichiometric coefficients were extracted from the literature directly or estimated based on the experimental conditions and other experimental results from the literature. For example, the biofilm thickness (625 µm) and CO_2_ permeability in gas-permeable PTFE were estimated based on the experimental conditions and results of Guo et al. (2019) [28] and Zhang et al. (2003) [41], while the biofilm density (50 kg dry biomass/m^3^) and PTFE membrane skin layer thickness (50 µm) were assumed, based on the typical values of these parameters in the literature [42], but were not measured directly. These values might not perfectly fit into the experimental conditions of the study by Guo et al. (2019) [28]. Thus, discrepancies between the modelling results and experimental results are not surprising. However, a reasonably good agreement (an error between 14.1 and 56.1% in most cases) between the modelling results and the experimental results from the literature was achieved. To improve the accuracy of the modelling prediction, the values of the parameters used in the mathematical models should be calibrated using the same experimental conditions. Furthermore, performing better-controlled experiments of MC-MBPBRs to acquire all the needed parameters as input values to the mathematical model would help further improve the accuracy of the model predictions.

## 4. Conclusions

A set of mathematical equations was developed to model the diffusion and reaction of CO_2_ and nutrients (N and P) in a flat-sheet gas-permeable hydrophobic membrane-attached microalgal biofilm. This study demonstrated that process conditions, such as CO_2_ concentration in the bulk gas phase, TN and/or TP concentrations in the bulk wastewater stream, and the biofilm thickness, had a significant impact on the process efficiency. An optimal biofilm thickness existed for maximum CO_2_, TN, and TP fluxes into the biofilm for bioreaction under each set of process conditions, implying the importance of biofilm thickness control in the operation of MC-MBPBRs. The results showed that mathematical modelling can be an effective and powerful tool for the optimal design and operation of the novel and emerging MC-MBPBR technology for wastewater treatment. The proposed mathematical models can be used to optimize the operation and to diagnose the operational problems of MC-MBPBRs, thus saving time and energy costs in wastewater treatment.

The proposed mathematical models can serve as a powerful tool for designing the optimal operation parameters of MC-MBPBRs to achieve maximum biofilm productivity and nutrient (N and P) removal. The simplified mathematical models developed here are potentially applicable to MC-MBPBRs having a thin biofilm thickness to minimize the impact of light attenuation through optimal MC-MBPBR system design to promote light penetration and uniform light distribution. Gas-permeable bifunctional lighting/supporting membrane systems can be designed to minimize the impact of light attenuation by using porous transparent membranes [54]. Periodical biofilm removal can be designed to maintain optimal biofilm thickness for optimal light transmission. Furthermore, dual light sources (one from the membrane side and the other from the biofilm–water interface side) can be used in MC-MBPBRs to achieve a greater biofilm thickness for obtaining light from both sides of the biofilm. Moreover, a flashlight LED device can be incorporated into the MC-MBPBR system design to minimize the impact of light attenuation [55]. From the nutrient dosing strategy point of view, the mathematical models are potentially applicable for the optimal design and operation of MC-MBPBRs for treating secondary effluent of domestic wastewater treatment; the low TN and TP loading would prevent rapid development and overgrowth of thick microalgae biofilm and thus maintain a thin biofilm with a minimal light attenuation effect.

Future modelling studies should include other important factors, such as potential light attenuation, CO_2_ dissociation (CO_2_/HCO_3_^−^/CO_3_^2−^), and dynamic biofilm thickness (considering both biofilm growth and detachment), to establish more comprehensive mathematical models; well-controlled lab-scale MC-MBPBR experiments—including the accurate measurements of biofilm density and thickness, microbial kinetic constants, stoichiometric coefficients, and homogeneous biofilm thickness development on membrane surfaces—should also be conducted to validate the modelling results.

## Figures and Tables

**Table 1:** Values of microbial kinetic parameters, stoichiometric coefficients, and process parameters used in this modelling study of an MC-MBPBR (25 °C).

Symbol of Parameter	Definition	Value at 25 °C	Unit	References and Notes
*D*_CO2-_ *_eff_*	Effective diffusivity of CO_2_ in biofilm	9.40 × 10^−10^	m^2^/s	[34] (estimated by using *D_eff_*/*D_aq_* = 0.5)
*D_N-eff_*	Effective diffusivity of NO_3_^−^-N ions in biofilm	1.25819 × 10^−9^	m^2^/s	[27,35] (estimated by *D_eff_*/*D_aq_* = 0.65)
*D_P-eff_*	Effective diffusivity of HPO_4_^2−^ ions in biofilm	5.72 × 10^−10^	m^2^/s	[35] (estimated by using *D_eff_*/*D_aq_* = 0.65)
*D*_CO2-_ *_H_*_2*O*_	Diffusivity of CO_2_ in water	1.880 × 10^−9^	m^2^/s	[36] (experimental results)
*D_N-H_*_2*O*_	Diffusivity of NO_3_^−^-N in water	1.93568 × 10^−9^	m^2^/s	[27] (converted from 20 °C to 25 °C)
*D_P-H_*_2*O*_	Diffusivity of HPO_4_^2−^ ions in water	8.80 × 10^−10^	m^2^/s	[35] (experimental results)
*H*	Henry’s law constant of CO_2_	0.032258	atm·m^3^/mol	[37]
*K_CO_*_2_	Monod half-saturation constant of CO_2_	4.4	g CO_2_/m^3^	[27,38]
*K_N_*	Monod half-saturation constant of N ions	7.1129	g N/m^3^	[39] (calibrated)
*K_P_*	Monod half-saturation constant of P ions	3.3906	g P/m^3^	[39] (calibrated)
*L*	Flat-sheet PTFE membrane thickness	5.0 × 10^−5^	m	*L* = 50 μm (assumed, based on commercially available PTFE membranes)
*L_b_*	Thickness of stagnant boundary layer of water	3.5 × 10^−3^	m	Estimated based on the experimental conditions of [28] and the method of estimating the stagnant boundary thickness of [40]
*L_f_*	Thickness of microalgal biofilm	0–1.0 × 10^−3^	m	Process parameter (*L_f_* = 0–1000 µm)
*P_m_*	Permeability of CO_2_ in PTFE membrane	7.0 × 10^−7^	mol CO_2_/atm·s·m	Estimated from the experimental data of [41]
*P_0_*	Partial pressure of CO_2_ in the gas phase in the lumen side of membrane at 1.0 atm	0.00034 (air), 0.01, 0.03, 0.05, 0.10	atm	Process parameter
*S_CO_*_2_	Concentration of CO_2_ in biofilm	-	g CO_2_/m^3^	Process parameter
*S_N-b_*	Concentration of N ions in the bulk phase of wastewater	247.1, 15	g N/m^3^	Influent N concentrations ([28] and assumption)
*S_N_*	Concentration of N ions in biofilm	-	g N/m^3^	Process parameter
*S_P-b_*	Concentration of P ions in the bulk phase of water	7.12, 2.5	g P/m^3^	Influent P concentrations ([28] and assumption)
*S_P_*	Concentration of P ions in biofilm	-	g P/m^3^	Process variable
x	Distance from the membrane and biofilm interface (x = 0): x = 0-*L_f_*	0–1.0 × 10^−3^	m	*L_f_* = 0–1.0 × 10^−3^ m (assumed)
*X_m_*	Microalgal concentration in biofilm	50,000	g microalgae/m^3^	50 kg/m^3^ (assumed, based on the typical range (39.5–85.9 kg/m^3^) of microalgal biofilm density in the literature [42])
*Y_M-CO_*_2_	CO_2_ consumption rate of microalgae	2.1824	g CO_2_ consumed/g microalgae produced	[43]
*Y_M-N_*	Microalgal cell yield based on N consumption	54.0	g microalgae produced/g N removed	Calibrated from the typical values (13–54 g microalgae produced/g N removed) estimated from experimental data of [30]
*Y_M-P_*	Microalgal cell yield based on P consumption	114.695	g microalgae produced/g P removed	[39]
*µ_max-m_*	Maximum specific growth rate of microalgae	1.94 × 10^−5^	1/s	[33,38] (assumed 1.68 d^−1^ from the typical range (0.984–2.0 d^−1^) of *µ_max-m_* in multiple-factor Monod equations in the literature [33])

**Table 2:** Comparison of the modelling and experimental results of CO_2_ and TN fluxes into the biofilm of an MC-MBPBR (Temperature = 25 °C).

Experimental Conditions * (*S_N-b_* = 247.1 mg N/L; *S_P-b_* = 7.12 mg P/L; Gas Phase CO_2_ = 10%)			Calculated Flux Results Based on Other Experimental Results ***		Flux Results by Modelling (*Y_M-N_* = 82 g Microalgae Produced/g N Removed (Calibrated))		Error % = (Expt. Result − Model. Result)/Expt. Result	
Gas flow rate	Liquid flow rate	Stable biofilm thickness ** (approx.)	CO_2_ flux into biofilm	TN flux into biofilm	CO_2_ flux into biofilm	TN flux into biofilm	CO_2_ flux error %	TN flux error %
mL/min	mL/h	µm	g CO_2_/m^2^·d	g N/m^2^·d	g CO_2_/m^2^·d	g N/m^2^·d	-	-
3	1	625	52.55	0.200	32.11	0.178	38.8	11.0
3	2		56.613	0.178			43.3	0.0
3	3		56.147	0.148			42.8	−20.3
3	4		56.147	0.214			42.8	16.8
1	2		34.58	0.198			7.14	10.1
3	2		55.283	0.171			41.9	−4.1
5	2		62.481	0.153			48.6	−16.4
average		625	53.4	0.180	32.11	0.178	39.9	1.27

* Data from Guo et al. (2019) [28]; ** estimated based on the biofilm mass values and experimental conditions from Guo et al. (2019) [28]; *** calculated from other experimental results of Guo et al. (2019) [28].

## Data Availability

Data from this study are available upon request.

## Supplementary Materials

The following supporting information can be downloaded at https://www.mdpi.com/article/10.3390/membranes15090269/s1: Supplementary Materials-Modelling of Diffusion and Reaction in MC-MBPBR-Liu and Liao.

## Abbreviations

The following abbreviations are used in this manuscript:

COD	chemical oxygen demand
DIC	dissolved inorganic carbon
MC-MBPBR	membrane carbonated microalgal biofilm photobioreactor
PTFE	polytetrafluoroethylene
TN	total nitrogen
TP	total phosphorus
